# Supplementary material for: A LON-ClpP Proteolytic Axis Degrades Complex I to Extinguish ROS Production in Depolarized Mitochondria
Source: Cell Rep. 2016 Dec 6;17(10):2522–31. doi: 10.1016/j.celrep.2016.11.027 (PMC5177631; doi:10.1016/j.celrep.2016.11.027)
Supplement: Document S1. Supplemental Experimental Procedures and Figures S1–S3 [file mmc1.pdf]

Cell Reports, Volume *17*

Supplemental Information

A LON-ClpP Proteolytic Axis Degrades Complex I to Extinguish ROS Production in Depolarized Mitochondria

Kenneth Robert Pryde, Jan Willem Taanman, and Anthony Henry Schapira

Supplementary information

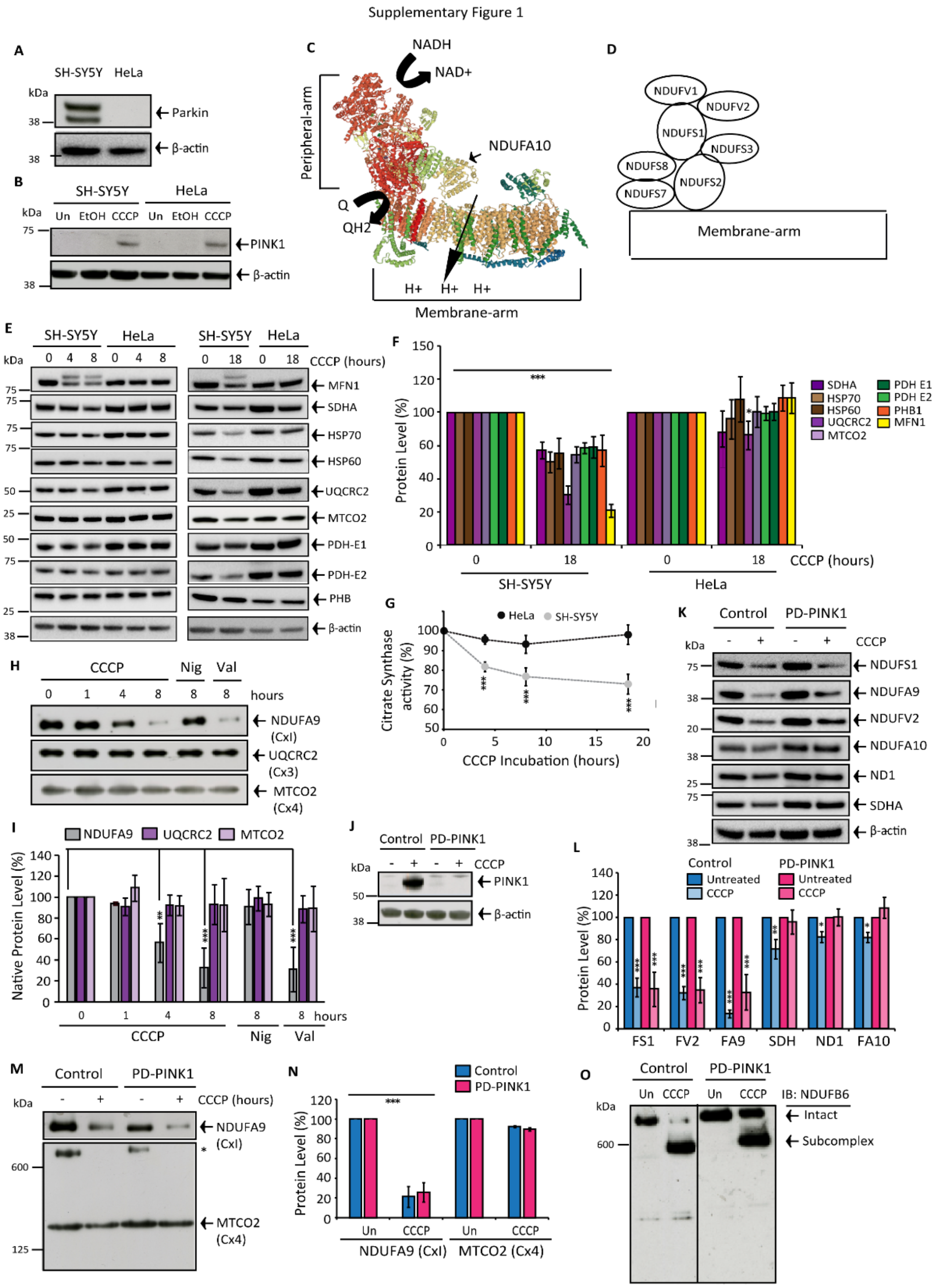

Supplementary Figure 1. Related to Figure 1: Complex I architecture, mitochondrial marker protein abundance during CCCP treatment and complex I degradation in control/PD-PINK1 fibroblasts. **(A-B)** SH-SY5Y and HeLa cells ± 18 hours CCCP or ethanol (EtOH). **(C)** High resolution structure of complex I from *Bos taurus* determined by single-particle cryo-microscopy. Protein Data Bank code 4UQ8.pdb. **(D)** Cartoon depiction of peripheral-arm subunit configuration. **(E-F)** SH-SY5Y and HeLa cells ± CCCP treatment (n=4-5). **(G)** Citrate synthase activity in SH-SY5Y and HeLa cells ± CCCP. **(H-I)** Native-PAGE. SH-SY5Y cells ± CCCP, nigericin (Nig) or valinomycin (Val) (n=3). **(J)** Control and PD-PINK1 fibroblasts ± 18 hours CCCP. **(K-L)** Control and PD-PINK1 fibroblasts ± CCCP (n=4). **(M-N)** Native-PAGE. Control and PD-PINK1 fibroblasts ± 18 hours CCCP. * indicates remnant complex I staining (n=3). **(O)** Native-PAGE. Control and PINK1-PD fibroblasts ± 18 hours CCCP. All data presented as mean + SDM.

Supplementary Figure 2

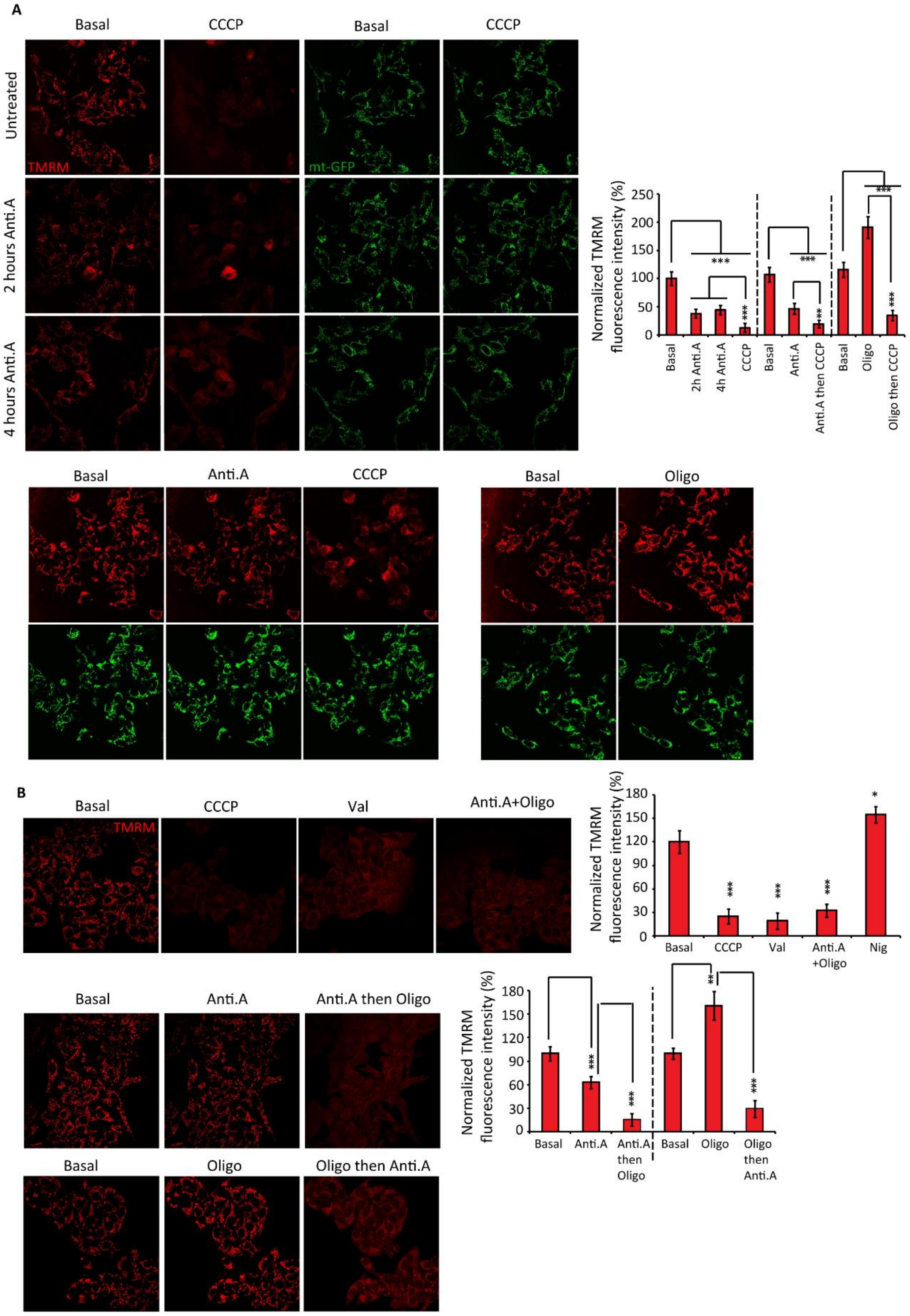

Supplementary Figure 2. Related to Figure 2: Effect of mitochondrial toxins on TMRM$^+$ fluorescence. **(A-B)** TMRM$^+$ fluorescence in SH-SY5Y cells expressing mitochondrial-targeted GFP **(A)** or HeLa cells **(B)**. CCCP, oligomycin (Oligo), valinomycin (Val), nigericin (Nig), antimycin A (Anti.A) and Anti.A+Oligo added as indicated. Representative images are taken from the steady-states for respective conditions (n=3). In **(B)** the upper panels/images reflect the average steady-state fluorescence after toxin addition in separate experiments (e.g. Val was not added after CCCP). All data presented in figure are mean values + SDM.

Supplementary Figure 3

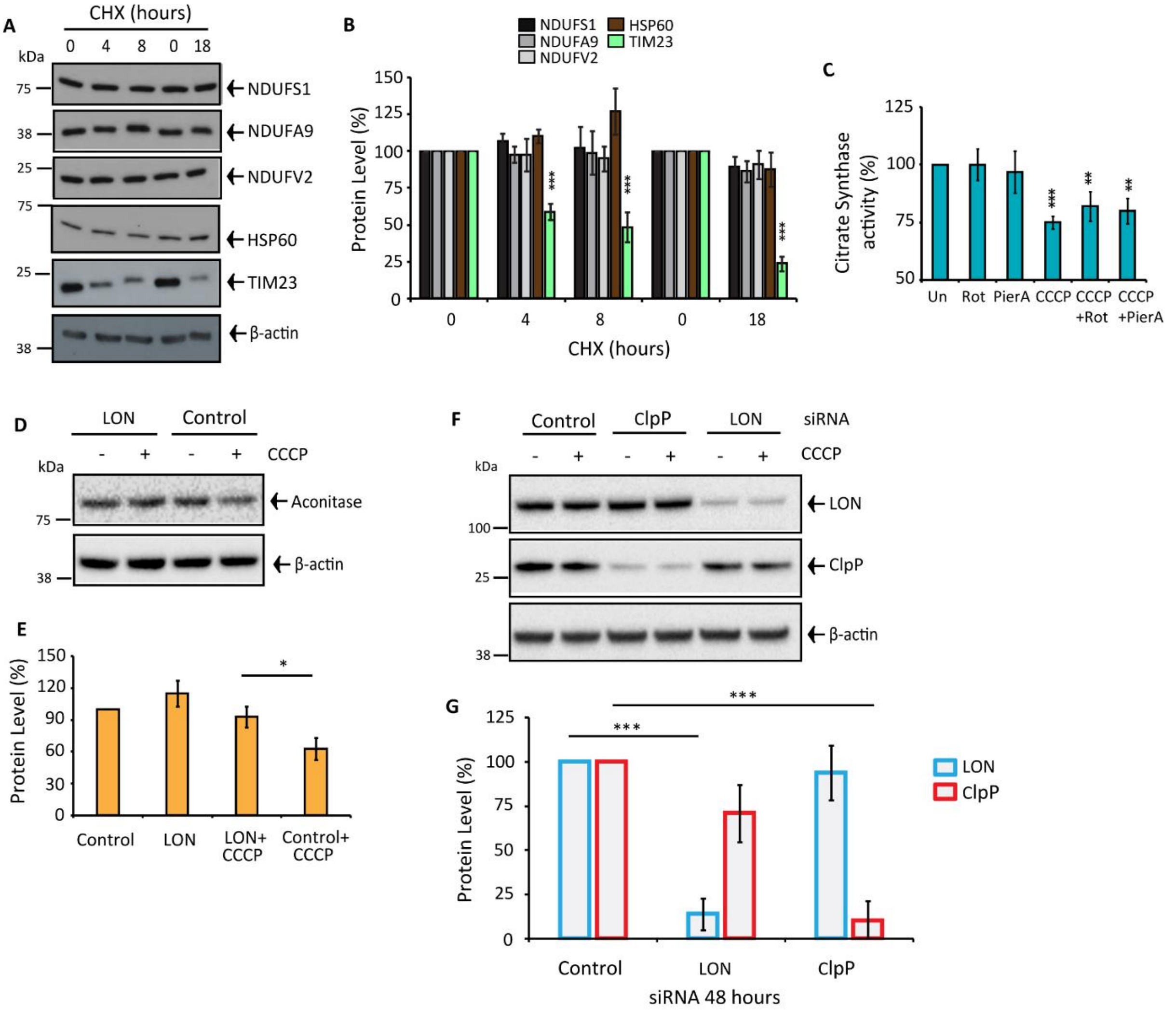

Supplementary Figure 3. Related to Figures 2, 3 and 4: Cycloheximide does not affect complex I level and the LON/ClpP silencing efficacy.

(A-B) HeLa cells ± cycloheximide (CHX) (n=3). **(C)** Citrate synthase activity. SH-SY5Y cells ±18 hours CCCP ± rotenone (Rot) or piericidin A (Pier.A) (n=4). **(D-E)** Aconitase immunoblots and quantification. HeLa cells ± 48 hours LON silencing followed by 8 hours CCCP (n=3). **(F-G)** ClpP (n=4) and LON (n=4) silencing in HeLa cells (48 hours) ± 8 hours CCCP. All data presented in figure are mean values + SDM.

Reagents and antibodies

All reagents/chemicals were purchased from Sigma-Aldrich unless otherwise stated. Antibodies were as follows: rabbit anti-NDUFS1 (Abcam - ab169540), rabbit anti-NDUFV1 (ProteinTech - 11238-1-AP), mouse anti-NDUFS2 (Abcam - ab192022), mouse anti-NDUFA9 (Abcam - ab14713), rabbit anti-NDUFV2 (ProteinTech - 15301-1-AP), mouse anti-NDUFB6 (Abcam - ab110244), mouse anti-NDUFB8 (Abcam - ab110242), rabbit anti-ND1 (ProteinTech - 19703-1-AP), rabbit anti-NDUFA10 (Abcam - ab174829), mouse anti-MFN1 (Abcam - ab57602), mouse anti-SDHA (Abcam - ab14715), mouse anti-UQCRC2 (Abcam - ab203832), mouse anti-PDH E1 (ProteinTech - 66119-1-Ig), rabbit anti-PDHA2 (Abcam - ab107015), mouse anti-β-actin (Abcam - ab8226), mouse anti-HSP60 (BioLegend – 681502), mouse anti-HSP70 (BioLegend - 648002), rabbit anti-PHB1 (Cell Signaling – 2426S), rabbit anti-LONP1 (ProteinTech - 15440-1-AP), rabbit anti-ClpP (Abcam - ab124822), rabbit anti-LC3B (D11) (Cell Signaling - 3868), rabbit anti-PINK1 (Novus Biologicals - BC100-494), mouse anti-Parkin (Santa Cruz Biotechnology - sc-32282), mouse anti-MTCO2 (Abcam - ab110258), mouse anti-TIM23 (BD Biosciences - 611222); rabbit anti-aconitase2 (ProteinTech 11134-1-AP).

Cell treatments

20 µM CCCP was added to SH-SY5Y or HeLa (S3) cells and 40 µM added to fibroblasts. When required, 1 µM valinomycin (Abcam), 0.5 µM nigericin (Abcam), 10 µM rotenone (Abcam), 10 µM piericidin A (Cayman Chemicals), 10 µM antimycin A (Abcam), 5 µM oligomycin (Abcam), 10 mM NAC (Sigma), 25 µM MG-132 (Abcam), 0.2 µM bafilomycin (Abcam) and 50 µg mL^{-1} cycloheximide (Sigma) was used.

Human primary fibroblasts

Control fibroblasts were from a 52 year old male and the PD-PINK1 deficient fibroblasts were cultured from a 48 year old male carrying a homozygous frameshift deletion in PINK1 (c.261_276del16;p.Try90Leufsx12).

Blue native-PAGE and 'in-gel' complex I activity measurements

Cell pellets were solubilised in 50 µL 1 M 6-aminocaproic acid, 50 mM bistris (pH 7 (HCl), 1.5 % n-Dodecyl β-D-Maltopyranoside (Anatrace) supplemented with 1 mM PMSF, 1 µg mL^{-1} of pepstatin and 1 µg mL^{-1} of leupeptin. Clarified lysates were added to Serva blue G loading buffer (5 % Serva blue G in 1 M 6-aminocaproic acid) with approximately 20-25 µg protein loaded onto non-commercial 3-12% acrylamide gels (acrylamide:bis-acrylamide, 0.5 mM 6-aminocaproic acid, 50 mM bistris (pH 7)) (Schägger, 1995). Complexes were resolved at 4 mA/gel for approximately 70 minutes. Cathode buffer comprised 50 mM tricine, 15 mM bistris, 0.02 % Serva blue G and the anode buffer 50 mM bistris. For immunoblot analysis, wet transfers were performed at 100 V for 1 hour and membranes subsequently processed as standard.

For 'in-gel' activity measurements the cathode buffer was replaced after 12 minutes with 50 mM tricine, 15 mM bistris without Serva blue. After ~ 65 minutes the gels were stained with a 2 mM Tris (pH 7.4, HCl), 150 µM NADH and 3 mM nitroblue tetrazolium for 1-2 hours at 37 °C. The assay buffer was exchanged with 10 % acetic acid-40 % methanol followed by distilled water. Gels were scanned and intensity quantified using Image J software.

Mitochondrial isolation

HeLa cells were re-suspended in ice cold 250 mM sucrose, 1 mM EDTA, 10 mM Tris (pH 7.4 (HCl)) buffer supplemented with 1x EDTA-free protease-phosphatase inhibitors, ruptured via mechanical homogenisation (1500 RPM, 20 strokes) and then pelleted (1500 g, 10 mins, 4 °C). Supernatants were collected and pellets re-homogenised (1500 RPM, 20 strokes), subsequently clarified (at 1500 g, 10 mins, 4 °C) and supernatants pooled. Mitochondria were pelleted (11800 g, 12 minutes, 4 °C), twice washed in isolation buffer, and pellets finally re-suspended in 50-125 µL isolation buffer.

Citrate synthase, aconitase, respiratory complexes I-IV and H_2O_2 activity measurements

Citrate synthase activity: 10-40 µg mL^{-1} of 1 % Triton X-100-PBS clarified cell lysate was added to 200 µM DTNB, oxaloacetate and acetyl-CoA (50 mM Tris pH 8.0). The steady-state formation of TNB was measured at 412 nm.

Aconitase activity: 1-2 mg mL^{-1} of cell lysate or 0.1-0.25 mg mL^{-1} mitochondrial lysate was incubated with 200 µM $NADP^+$, 1 mM $MgCl_2$, 500 µM sodium citrate and exogenous isocitrate dehydrogenase (50 mM Tris pH 8.0). Steady-state rates of NADPH formation were monitored at 340 nm.

Respiratory complexes I-IV: Isolated mitochondria from cells ± CCCP treated were re-suspended in 320 mM sucrose, 1 mM K^+-EDTA, 10 mM Hepes (pH 7.4, HCl) and snap frozen 3x. Complex IV activity (550 nm) = 10 mM KPi buffer (pH 7.0) and 50 µM reduced cytochrome *c*, 30°C. Complexes II-III (550 nm) = 0.1 M KPi buffer (pH 7.4), 0.3 mM EDTA, 1 mM KCN, 20 mM succinate, 2.5 mg mL^{-1} BSA, 100 µM oxidised cytochrome *c* ± antimycin A, at 30°C. Complex I (340 nm) = 20 mM KPi buffer (pH 7.2), 1 mM $MgCl_2$, 1 mM KCN, 2.5 mg ml^{-1} BSA, 150 µM NADH, 50 µM CoQ_1 ± rotenone, 30°C.

Mitochondrial H_2O_2 production: Isolated mitochondria were snap frozen 3x (each time clarified (11800 g, 12 minutes, 4 °C)) and added to 80 µM NADH, 10 µM amplex red and 4 mg mL^{-1} HRP (250 mM sucrose, 10 mM Tris pH 7.5). Catalase-dependent steady-state rates of resorufin formation were monitored at 557 nm.

Respiration and glycolysis measurements in cultured cells

HeLa cells were seeded into XFp miniplates (Seahorse Bioscience) at $2.5x10^4$ and cultured overnight. Subsequently, cells were washed and incubated with XF base medium supplemented with exogenous 10 mM glucose, 1 mM pyruvate and 2 mM glutamine (pH 7.4 (NaOH)) and placed in a CO_2-free incubator at 37°C for 60 minutes. Oxygen consumption rates and extracellular acidification rates were measured in a XFp extracellular flux analyser (Seahorse, Agilent Technologies). Rates were adjusted if necessary following protein determination (BCA) and the antimycin A+rotenone insensitive rates were subtracted.

Obtaining and quantifying immunofluorescence and live-cell imaging data

For fixed cells images were acquired using an inverted Zeiss LSM 510 confocal microscope and a 63x oil-immersion objective. Alexa 594 was excited using the 543 nm laser line (HeNe laser) and fluorescence detected with a 560 nm long-pass filter. Alexa 488 was excited using a 488 nm laser line (Argon laser) and the fluorescence measured between 510 nm to 550 nm. Laser power and other settings were conserved during replicate experiments. Image J was used to quantify maximal fluorescence intensity (following background subtraction) in more than 15-25 cells per repeat experiment (n=3) and in 3-5 different fields of view. Data is presented as the average maximal fluorescence and in arbitrary units.

For live cell imaging GFP was excited using the 488 nm laser line (Argon laser) and emission measured between 510-550 nm, and $TMRM^+$ excited using the 543 nm laser line (HeNe laser) with emission measured using a 560 nm long-pass filter. MitoSOX was exited at 380 nm and emission followed using a 560 nm long-pass filter. NAD(P)H autofluorescence was excited at 351 nm and measured between 375-470 nm. Fields of view comprising 20-40 cells were selected and Z-projected (n = 3). Imaging was performed using a Zeiss LSM 510 (NAD(P)H autofluorescence and mitoSOX) or Nikon confocal microscope (GFP and $TMRM^+$) and either a 63x oil-immersion objective (GFP, mitoSOX and $TMRM^+$) or a 60x water-immersion objective (NAD(P)H). Average intensities were determined across the Z-stack throughout the time course (ImageJ). Data is presented as the percentage change from the initial measurement or fluorescence intensity (arbitrary units). All toxins were added in a HBSS solution and when necessary comprised the equivalent concentration of fluorescent dye.
