# Supplementary material for: A LON-ClpP Proteolytic Axis Degrades Complex I to Extinguish ROS Production in Depolarized Mitochondria
Source: Cell Rep. 2016 Dec 6;17(10):2522–31. doi: 10.1016/j.celrep.2016.11.027 (PMC5177631; doi:10.1016/j.celrep.2016.11.027)
Supplement: Document S2. Article plus Supplemental Information [file mmc2.pdf]

Cell Reports

Report

A LON-ClpP Proteolytic Axis Degrades Complex I to Extinguish ROS Production in Depolarized Mitochondria

Graphical Abstract

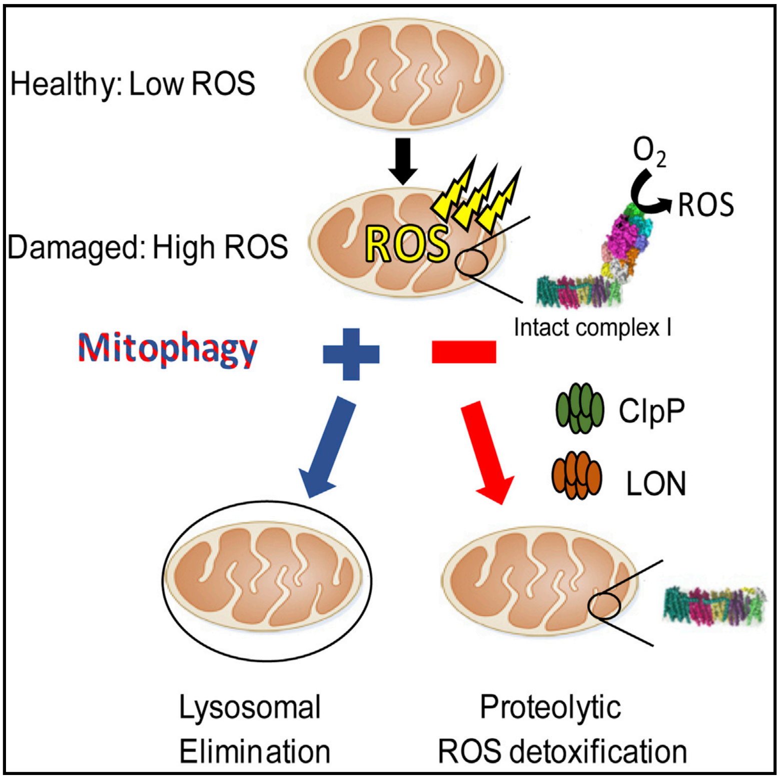

Highlights

- Complex I is selectively vulnerable to proteolysis in depolarized mitochondria
- Complex I degradation extinguishes high ROS if mitophagy fails
- LON and ClpP bind and degrade complex I after mitochondrial stress induction
- Complex I abundance is coupled to mitochondrial homeostasis

Authors

Kenneth Robert Pryde,
Jan Willem Taanman,
Anthony Henry Schapira

Correspondence

kp296@leicester.ac.uk

In Brief

Damaged mitochondria are thought to accumulate if mitophagy is impaired and to cause Parkinson's disease by continuously generating high levels of toxic reactive oxygen species (ROS). Pryde et al. show instead that proteolytic quality control ensues in these organelles to confine and diminish ROS by degrading the ROS-producing domain of complex I.

CrossMark

Pryde et al., 2016, Cell Reports *17*, 2522–2531
December 6, 2016 © 2016 The Author(s).
http://dx.doi.org/10.1016/j.celrep.2016.11.027

CellPress

OPEN ACCESS
CellPress

Cell Reports
Report

A LON-ClpP Proteolytic Axis Degrades Complex I to Extinguish ROS Production in Depolarized Mitochondria

Kenneth Robert Pryde,[1,2,*] Jan Willem Taanman,[1] and Anthony Henry Schapira[1]
[1]Department of Clinical Neurosciences, Institute of Neurology, University College London, London WC1E 6BT, UK
[2]Lead Contact
*Correspondence: kp296@leicester.ac.uk
http://dx.doi.org/10.1016/j.celrep.2016.11.027

SUMMARY

Mitochondrial dysfunction is implicated in numerous neurodegenerative disorders and in Parkinson's disease (PD) in particular. PINK1 and Parkin gene mutations are causes of autosomal recessive PD, and these respective proteins function cooperatively to degrade depolarized mitochondria (mitophagy). It is widely assumed that impaired mitophagy causes PD, as toxic reactive oxygen species (ROS)-producing mitochondria accumulate and progressively drive neurodegeneration. Instead, we report that a LON-ClpP proteolytic quality control axis extinguishes ROS in depolarized mitochondria by degrading the complex I ROS-generating domain. Complex I deficiency has also been identified in PD brain, and our study provides a compelling non-genetic mechanistic rationale to explain this observation: intact complex I depletes if mitochondrial bioenergetic capacity is robustly attenuated.

INTRODUCTION

Parkinson's disease (PD) is the second most prevalent neurodegenerative disorder (Schapira, 2008). Mitochondrial dysfunction is implicated in most PD genetic variants and is also reported in sporadic PD, suggesting mitochondrial homeostasis and PD are connected (Schapira, 2008). For instance, brains from sporadic PD patients typically display a selective deficiency in mitochondrial complex I activity (via an uncertain mechanism), and they comprise mitochondrial/cellular oxidative damage (Schapira et al., 1989; Keeney et al., 2006; Parker et al., 2008). Mutations in PINK1 (a mitochondrial kinase) and Parkin (a cytosolic E3 ubiquitin ligase) are two autosomal recessive causes of PD (Kitada et al., 1998; Valente et al., 2004). PINK1-Parkin function cooperatively to degrade damaged/depolarized mitochondria via autophagy (mitophagy) (Narendra et al., 2010). PINK1 is the damage sensor; it is imported into the inner mitochondrial membrane (IMM) of polarized mitochondria and degraded. Mitochondrial depolarization terminates IMM import (Malhotra et al., 2013), so PINK1 rapidly accumulates in the outer mitochondrial membrane (OMM) and phospho-activates Parkin (Kane et al., 2014) to initiate mitophagy.

Mitochondria also can produce copious amounts of deleterious reactive oxygen species (ROS). Respiratory complex I has long been considered the predominant source, and complex I abundance parallels mitochondrial ROS-generating capacity (Lambert et al., 2010; Miwa et al., 2014). Oxygen is reduced to ROS via a side reaction with the NADH-reduced flavin cofactor (Pryde and Hirst, 2011). In isolated mitochondria, the rates of ROS formation accelerate significantly when inhibitors, mimicking molecular damage, impair respiration (Kushnareva et al., 2002). It is assumed that high ROS-generating damaged mitochondria accumulate if mitophagy fails and these organelles contribute to, or cause, PD via ongoing oxidative damage to affected neurons. Yet other than inference, the empirical basis supporting this orthodoxy is lacking. Little is known about the fate and properties of the organelles normally cleared by mitophagy; their fundamental ROS-generating capacities are not established or defined. It is also feasible that mitophagy-independent PINK1 and Parkin activities cause PD (Chung et al., 2001; Morais et al., 2014). Elucidating why PINK1-Parkin deficiency causes PD remains the outstanding question in the field.

Here we report that damaged mitochondria do not perpetually generate high ROS, as an intra-mitochondrial LON-ClpP proteolytic quality control axis functions to extinguish ROS in these organelles by degrading the ROS-producing domain of complex I.

RESULTS

Mitochondrial Complex I Is Selectively Degraded in Depolarized Mitochondria

To determine the fate of complex I in damaged organelles, we induced mitophagy using canonical methodology (Narendra et al., 2010), and we chemically depolarized mitochondria with the protonophore CCCP in SH-SY5Y and HeLa cells. Both cell types express PINK1 but HeLa cells do not express Parkin (Figures S1A and S1B). Mammalian complex I comprises 44 discrete subunits (Zhu et al., 2016). The peripheral arm protrudes into the matrix and catalyzes NADH oxidation, ROS production, and ubiquinone reduction. The membrane arm resides in the IMM and mediates proton translocation. Recently, high-resolution structures revealed the arrangement of all constituent subunits and redox/catalytic centers (Figure S1C) (Zhu et al., 2016).

 © 2016 The Author(s).
This is an open access article under the CC BY license (http://creativecommons.org/licenses/by/4.0/).

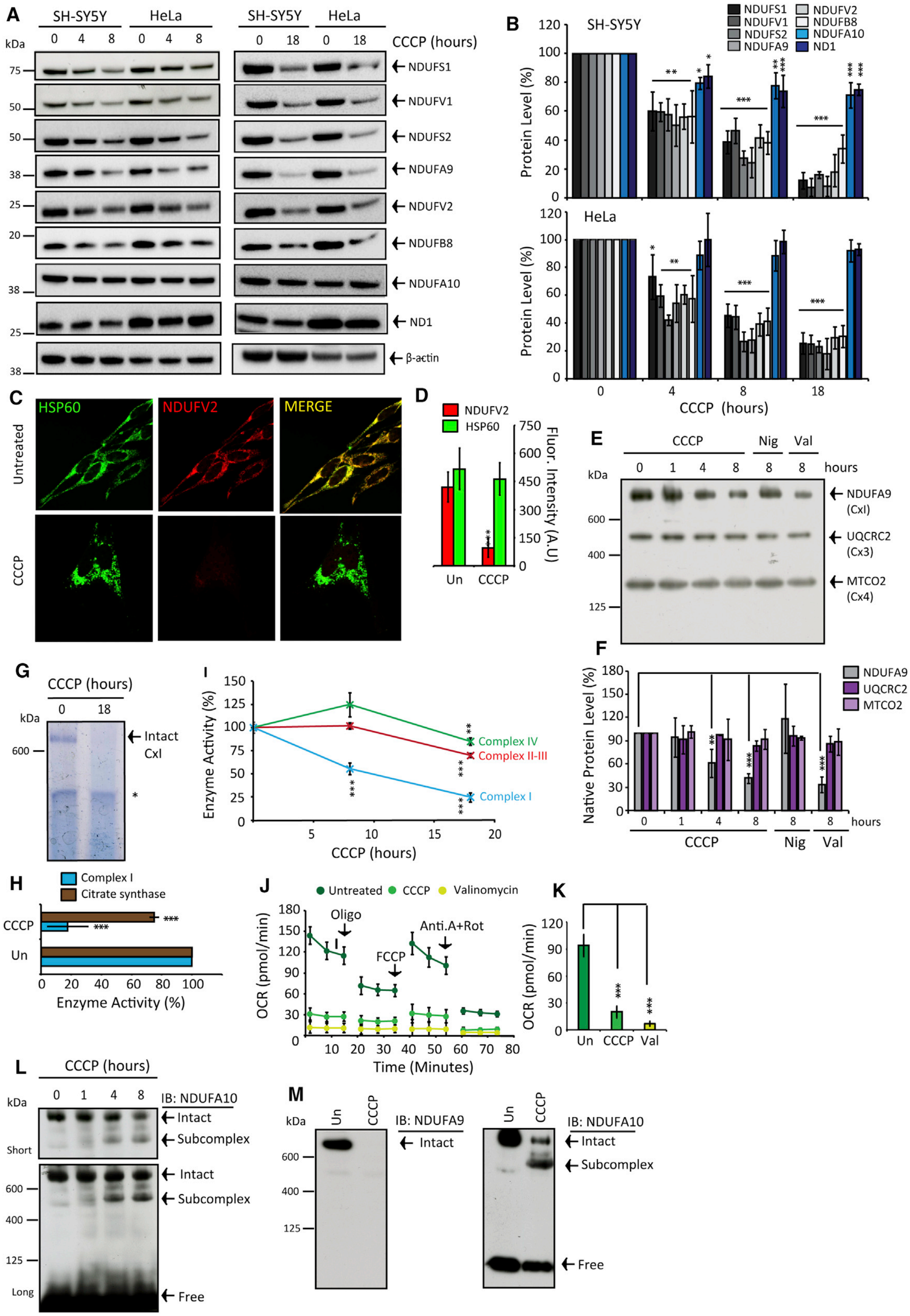

(legend on next page)

In both cell types, CCCP progressively diminished both catalytically core and accessory peripheral arm FV1, FV2, FS1, FS2, and FA9 subunits (core subunits depicted in Figure S1D); all decreased by ~35%–40%, 60%–70%, and 80%–95% following 4-, 8-, and 18-hr CCCP, respectively (Figures 1A and 1B). In contrast, two subunits residing in the membrane arm, FA10 and ND1, were maintained. However FB8 notably depleted, thus not all membrane arm subunits were preserved. Peripheral arm depletion exceeded the magnitude of loss ascribable to mitophagy. In SH-SY5Y cells, a cohort of matrix and IMM proteins (as well as ND1 and FA10) maximally reduced by 20%–30%; these were largely unaffected in HeLa cells (Figures S1E–S1G). A notable exception was the complex III subunit UQCRC2, which decreased by 60%–70% in SH-SY5Y cells only. Figures 1C and 1D show FV2 immunostaining (core subunit), but not HSP60, greatly diminished in CCCP-treated HeLa cells. Therefore, enduring depolarized mitochondria are devoid of the catalytically active peripheral arm.

Native-PAGE immunoblot analysis revealed that subunit depletion corresponds to intact complex I degradation (Figures 1E, 1F, and 1M; Figures S1H and S1I), and both flavin and Q-site activities robustly diminished (Figures 1G–1I). Neither the amount nor size of complexes III and IV were affected during depolarization (Figures 1E and 1F; Figures S1H and S1I), and, after 18 hr, complex II–IV activities decreased by only ~25% (Figure 1I). As expected, diminished complex I robustly impaired oxygen consumption rates (Figures 1J and 1K).

Degradative subcomplex intermediates were not observed when immunoblotting for peripheral arm subunits, but a large complex emerged when probing for FA10 (Figures 1L and 1M) and the molecular weight shift was commensurate with membrane arm. Subcomplex abundance increased inversely to declining intact complex I levels (Figures 1L and 1M). Desynchronized peripheral and membrane arm degradation is perhaps not surprising considering distinctive modules assemble into the final configuration (Koopman et al., 2010). Finally, we confirmed that PINK1 is not requisite for CCCP-induced peripheral arm turnover (Figures S1J–S1O). Taken together, the peripheral arm is selectively vulnerable to proteolysis after depolarization independently of PINK1/Parkin/bulk mitophagy.

$m\Delta_{\Psi}$ Dissipation Signals Peripheral Arm Degradation

What drives complex I degradation following mitochondrial depolarization? CCCP equilibrates protons across the IMM, thereby dissipating the membrane potential ($m\Delta_{\Psi}$) and pH difference (mΔpH) components of the proton-motive force (Δp), as well as terminating Δp-driven ATP synthesis and $m\Delta_{\Psi}$-driven protein import. We used nigericin (Nig; dissipates mΔpH, increases $m\Delta_{\Psi}$), valinomycin (Val; dissipates $m\Delta_{\Psi}$, increases mΔpH), oligomycin (Oligo; inhibits ATP synthase), and cycloheximide (CHX; inhibits cytosolic translation) to dissect how CCCP signals proteolysis.

CHX did not significantly alter steady-state complex I levels (Figures 2A and 2B; Figures S3A and S3B), but it greatly diminished short-lived TIM23 (Figures S3A and S3B). Therefore, peripheral arm turnover is a stimulated response to depolarization and not attributable to half-life kinetics. Figures S2A and S2B show only CCCP and Val quenched $TMRM^{+}$ (a $m\Delta_{\Psi}$ reporter) and induced peripheral arm loss (Figures 1E, 1F, and 2A–2D).

Blocking respiration with the complex III inhibitor antimycin A (Anti.A) only depleted complex I (Figures 2C–2G) and in-gel activity (Figures 2H and 2I) when combined with Oligo. Anti.A + Oligo quenched $TMRM^{+}$ similarly to CCCP (Figure S2B), whereas Anti.A alone partially lowered $TMRM^{+}$ (Figure S2B), i.e., sufficient $m\Delta_{\Psi}$ is maintained via ATP hydrolysis by ATP synthase to preserve complex I. The complex I Q-site inhibitor rotenone (Rot) essentially recapitulated the Anti.A effect (Figures 2E and 2F). However, we noted that the degradation efficacy of Rot + Oligo was approximately two times weaker compared to Anti.A + Oligo. To establish if Rot somehow impairs proteolysis, we included Rot alongside CCCP and found ~2-fold less peripheral arm depletion (Figures 2J and 2K; Figure S3C). Similar results were observed with an alternative Q-site inhibitor, piericidin A (Figures 2J and 2K; Figure S3C).

Activating Mitophagy Triggers Mitochondrial Oxidative Stress and Peripheral Arm Depletion

Next, we sought to determine if activating mitophagy elevates mitochondrial ROS production and to explore how complex I degradation affects ROS. Mitochondrial aconitase (mt-aconitase) activity is an established reporter of intra-mitochondrial ROS (Gardner et al., 1995). Here, aconitase activity reduced by 50%–60% during 15- to 30-min Anti.A ± Oligo treatment (Figures 3A and 3B). Cells express two aconitase isoforms compartmentalized in the matrix and cytosol (Gardner et al., 1995). We isolated mitochondria from cells at ±1-hr Anti.A treatment and found eradicated aconitase activity; thus, 55% of the total rate is attributable to mt-aconitase. Rot ± Oligo abolished mt-aconitase activity after ~1-hr treatment, and CCCP progressively inactivated mt-aconitase albeit at a slower rate (Figure 3A). Including the antioxidant NAC diminished CCCP degradative potency, suggesting ROS contributes to the orchestration of peripheral arm turnover (Figure 3C). Thereby, conditions that deplete the peripheral arm robustly augment mitochondrial ROS production.

Figure 1. Δp Dissipation Triggers Selective Complex I Peripheral Arm Proteolysis

(A and B) SH-SY5Y and HeLa cells after 4-, 8-, and 18-hr CCCP treatment (n = 5).

(C and D) HSP60 and NDUFV2 immunostained HeLa cells ± 18-hr CCCP treatment (n = 3).

(E and F) Native-PAGE complex I, III, and IV levels, following 0- to 8-hr CCCP or 8-hr nigericin (Nig) or valinomycin (Val) (n = 3).

(G and H) Native-PAGE in-gel complex I activity in HeLa cells and quantification compared to citrate synthase activity ± 18-hr CCCP (*non-complex I reactive band; n = 3).

(I) Activity measurements in mitochondria isolated from HeLa cells ± CCCP (n = 3).

(J and K) Respiration rates in HeLa cells ± 8-hr CCCP or valinomycin pre-treatment. Oligomycin (Oligo), FCCP, and antimycin A + rotenone (Ant.A + Rot) were added as indicated (n = 4).

(L and M) Native-PAGE NDUFA9 and NDUFA10 immunoblots. HeLa cells ± 8- and 18-hr CCCP. All data are presented in the figure as mean + SDM.

See also Figure S1.

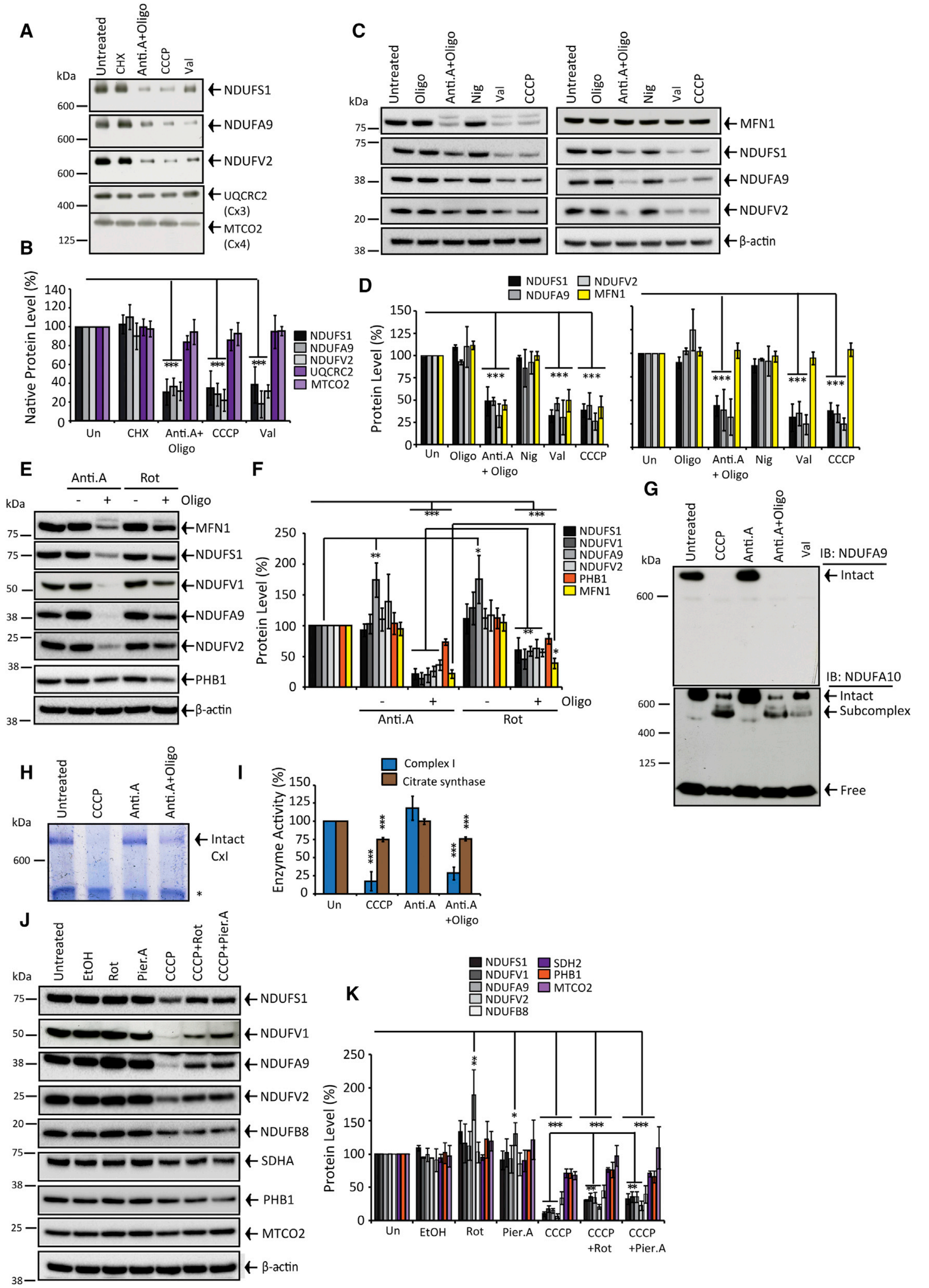

Figure 2. mΔ_{Ψ} Dissipation Is the Peripheral Arm Degradation Trigger

(A and B) Native-PAGE. HeLa cells ± 8-hr cycloheximide (CHX), antimycin A + oligomycin (Anti.A + Oligo), CCCP, or valinomycin (Val) treatment (n = 3).

(C and D) SH-SY5Y (left panel) and HeLa (right panel) cells ± 8-hr Oligo, Anti.A + Oligo, nigericin (Nig), Val, and CCCP (n = 3).

(legend continued on next page)

If oxidative stress initiates peripheral arm turnover, why does complex I maintain after 18-hr Anti.A or Rot treatment? We probed the precise kinetic relationship between Anti.A exposure and complex I stability. Remarkably, FS1, FV1, FA9, and FV2 levels declined by 20%–50% after 2- to 4-hr Anti.A, but subsequently they recovered to basal levels after 8 hr (Figures 3D and 3E); the recovery was blocked by CHX, and we observed concurrent upregulation of nuclear-encoded peripheral arm, but not membrane arm, transcript levels (Figures 3D–3F). The magnitude of transcript upregulation is consistent with previously determined mitochondria-nuclear retrograde signaling (Münch and Harper, 2016). Figures 2H and 2I confirm that intact complex I comprising reconstructed peripheral arm is catalytically active.

Is mitochondrial ROS production affected by complex I degradation? In live cells ROS measurements were limited to Anti.A treatments using mitoSOX (as equilibration of ROS reporters requires $m\Delta_\Psi$). Anti.A concurrently increased NAD(P)H autofluorescence, which decreases the complex I flavin reduction potential (Pryde and Hirst, 2011) (Figures 3G and 3H), and stimulated ROS formation by ~5- to 6-fold (Figures 3I and 3J). NAD(P)H intensity maintained during Anti.A treatment (Figures 3G and 3H), but ROS production lowered (Figures 3I and 3J) proportionally to peripheral arm depletion (Figures 3D and 3E). Furthermore, mitochondria fractionated from CCCP-treated cells generated significantly less NADH-induced ROS (Figure 3K). Mitochondrial ROS production progressively diminishes as complex I is degraded.

LON and ClpP Bind and Degrade the Peripheral Arm

Matrix and IMM proteins are degraded by an elaborate network of proteases (Quirós et al., 2015), including m-AAA as well as stress-activated LON and ClpP. The proteasome (Radke et al., 2008) and lysosome (Soubannier et al., 2012) also can mediate degradation.

Proteasomal or lysosomal inhibition did not affect peripheral arm turnover (Figures 4A–4D). Silencing LON impaired depolarization-induced FS1 (1.7×), FV1 (1.9×), FA9 (1.4×), and FV2 (2.0×) degradation, whereas silencing ClpP retarded FV1 (1.7×) and FV2 (3.1×) turnover (Figures 4E and 4F; Figures S3F and S3G), suggesting subunit-dependent proteolytic redundancy and exclusivity. Remnant turnover may reflect residual LON/ClpP activities or less efficient turnover pathways, and redundancy is a hallmark of the proteolytic network (Quirós et al., 2015). Silencing LON impaired mt-aconitase degradation in depolarized mitochondria (Figures S3D and S3E).

Do degradative proteases dock to intact complex I as part of the degradative cascade? We immuno-captured intact complex I via FB6; Figure 4G confirms co-precipitation with distal FV2 and FS1 subunits. LON and ClpP significantly enriched with precipitated intact complex I after 4-hr Anti.A treatment (Figure 4G). Reverse capture of LON also precipitated several of the degradable peripheral arm subunits (Figure 4H) (ClpP not unsuccessfully precipitated). Finally, Figure 2J revealed that rotenone impaired peripheral arm turnover, and Figure 4G shows rotenone diminished Anti.A-induced complex I and LON precipitation, suggesting Q-site-inhibited complex I is more resistant to proteolysis and protease binding.

DISCUSSION

Molecular mechanisms of PINK1-Parkin mitophagy have been studied intensely; however, the cause of PD is not understood. Here, we show that the widespread assumption that accumulating impaired mitochondria perpetually generate high toxic ROS levels requires reconsideration; quality control ensues to extinguish ROS by degrading complex I and, thus, constraining the putative toxicity phase (cartoon depiction in Figure 4I). Challenging studies are required to establish if the limited phase of high ROS is sufficient to cause PD and to determine if accelerating complex I degradation can be harnessed therapeutically.

We also provide evidence for an intriguing stress-induced complex I degradation-retrograde signaling-protein synthesis/import-repair cascade. Elucidating the orchestration of retrograde signaling and how repair is coordinated require further investigation, but presumably they enable respiration renewal by clearing/repairing damage-prone peripheral arm if the organelles retain some $m\Delta_\Psi$. ATP hydrolysis by ATP synthase generates $m\Delta_\Psi$ when the respiratory chain is impaired; thus, if glycolytic capacity is low (e.g., neurons [Almeida et al., 2001]), the repair pathway will fail, which might explain complex I deficiency in PD brains; thus far only mtDNA mutations had been considered (Kraytsberg et al., 2006). Intriguingly, in both PD human brains and PD models, as well as generic ROS-inducing conditions, peripheral arm subunits are selectively oxidized (including FS1, FS2, FV1, and FS6) (Keeney et al., 2006; Chinta et al., 2007; Danielson et al., 2011; Chouchani et al., 2013; Gorenkova et al., 2013). The LON-ClpP degradative axis now provides a plausible mechanistic foundation to bridge deficiency with oxidative damage.

Comprehensive in vitro experimentation is required to unravel the degradative mechanism. Elucidating degrons and inter-subunit degradative dependencies for all constituent peripheral arm subunits is experimentally daunting, considering single-point mutations can destabilize intact complex I (Varghese et al., 2015). Robust investigation will require structural determination of degradative intermediates combined with carefully executed mutagenesis. Previous studies revealed LON comprises specificity for both oxidized peptide and Fe-S clusters (Bota and Davies, 2002; Ngo and Davies, 2009; Bender et al., 2011; Kim et al., 2015). Thereby, complex I Fe-S clusters could form ROS sensors, with their susceptibility to oxidation signaling degradation

(E and F) SH-SY5Y cells ± 18-hr Anti.A ± Oligo or rotenone (Rot) ± Oligo (n = 3).
(G) Native-PAGE NDUFA9 or NDUFA10 immunoblots ± 18-hr Anti.A + Oligo, Anti.A, CCCP, or Val.
(H and I) Native-PAGE in-gel complex I activity (n = 3) and citrate synthase activity (cell lysates) (n = 5). SH-SY5Y cells ± 18-hr CCCP, Anti.A, or Anti.A + Oligo (*non-complex I reactivity).
(J and K) SH-SY5Y cells ± 18-hr Rot, piericidin A (Pier.A), CCCP, CCCP + Rot, or CCCP + Pier.A (n = 3). All data are presented in the figure as mean + SDM. See also Figure S2.

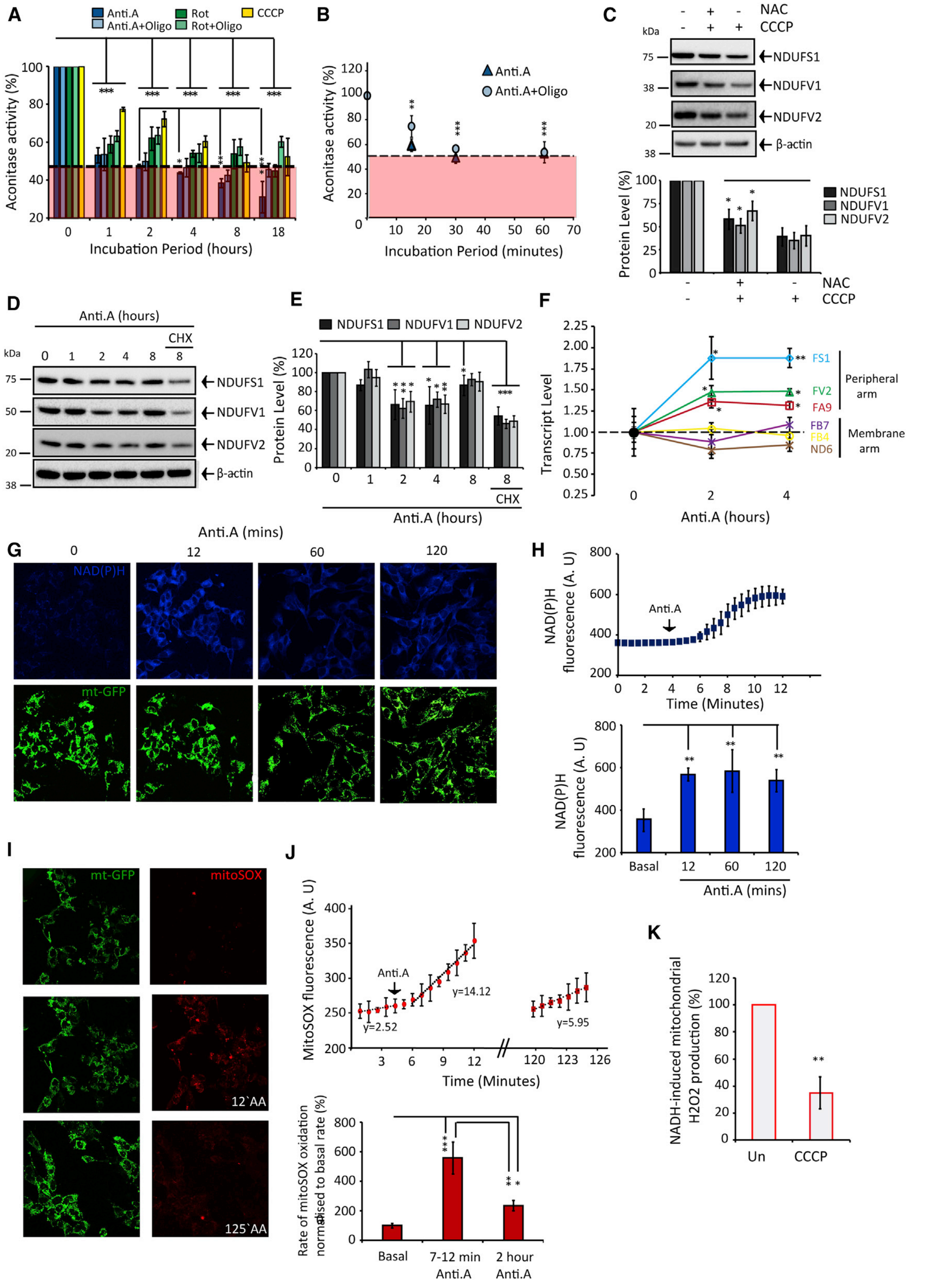

(legend on next page)

in combination with oxidized polypeptide or hydrophobic patches in denatured regions (Nishii et al., 2005; Ondrovicová et al., 2005).

The finding that Q-site-inhibited complex I is less vulnerable to turnover provides a mechanistic foundation for subsequent studies and indicates that the Q-site apparatus systemically controls the propensity of peripheral arm degron formation. Inactive complex I reverts to a distinctive deactive state (Zhu et al., 2016) in vitro (Kotlyar and Vinogradov, 1990) and in vivo (Maklashina et al., 2002). Deactive conformational change is largely restricted to Q-site architecture, exposing thiols in ND1, ND3, and FA9 (Galkin et al., 2008; Babot et al., 2014) to oxidation/redox modification (Gorenkova et al., 2013). Rotenone retards the deactive transition (Grivennikova et al., 1997) by probably binding peptide, which drives the conversion so complex I is jammed into a non-deactivate conformation. Accordingly, the deactive enzyme may form the degradative ground state by revealing degrons/exposing oxidizable peptide, which enables subsequent denaturation and damage contagion in a feedforward-like proteolytic mechanism. Considering rotenone- and MPP- (another Q-site inhibitor) treated mice exhibit parkinsonism, a convolution of defective respiration and suppression of complex I degradation may contribute.

In conclusion, our study establishes a fundamental connectivity between mitochondrial homeostasis and complex I. Previously, complex I deficiency in diseases such as PD has been considered in the context of genetic defects in constituent subunits and assembly factors. We now show complex I stability is governed by an additional non-genetic regulatory dimension. We provide a conceptual framework that couples complex I activity/abundance with perturbed cellular homeostasis. For instance, disease-associated pathways that trigger systemic or local depolarization of the mitochondrial pool, such as augmented nitric oxide during endoplasmic reticulum (ER) dysfunction or excitotoxicity, should induce peripheral arm depletion.

EXPERIMENTAL PROCEDURES

Cell Culture

HeLa (S3) and fibroblasts were cultured in DMEM Glutamax (Life Technologies) and SH-SY5Y cells in DMEM/F12 (Life Technologies) supplemented with 10% fetal bovine serum (FBS, Life Technologies), 1 mM pyruvate (Life Technologies), non-essential amino acids (Life Technologies), and penicillin/streptomycin. Cells were cultured in a 37°C, 5% CO_2 humidified atmosphere.

SDS-PAGE and Immunoblots

1% Triton X-100 extracts (supplemented with 1× EDTA-free protease/phosphatase inhibitors, Pierce) were equally loaded (bicinchoninic acid [BCA] protein assay), DTT reduced, resolved using SDS-PAGE (pre-cast 4%–12% gels; Novex, Life Technologies), and transferred (wet transfer) onto polyvinylidene fluoride (PVDF) membranes (GE Healthcare, Amersham Hybond, 0.45 μm). Membranes were blocked and then incubated with primary antibody overnight (4°C), followed by 1:2,000 horseradish peroxidase (HRP)-conjugated secondary antibodies (Dako) for 1 hr. Enhanced chemiluminescence (ECL) Clarity chemiluminescent substrate (Bio-Rad) was used to detect immunoreactive proteins by the ChemiDoc MP (Bio-Rad) or X-ray film. Unless otherwise stated, all immunoblots are adjusted for β-actin level and normalized to untreated samples (100%).

Blue Native-PAGE and in-Gel Complex I Activity Measurements

Cell pellets were solubilized in 50 μL 1 M 6-aminocaproic acid, 50 mM bistris (pH 7, HCl), and 1.5% n-Dodecyl β-D-Maltopyranoside supplemented with protease inhibitors. Clarified lysates were added to Serva blue G loading buffer and loaded onto non-commercial 3%–12% acrylamide gels (Schägger, 1995). Complexes were sufficiently resolved and membranes subsequently processed as standard.

For in-gel activity measurements, the cathode buffer was replaced after 12 min with 50 mM tricine and 15 mM bistris without Serva blue. After 65 min, the gels were stained with a 2 mM Tris (pH 7.4, HCl), 150 μM NADH, and 3 mM nitroblue tetrazolium for 1–2 hr at 37°C. The assay buffer was exchanged with 10% acetic acid-40% methanol followed by distilled water. Gels were scanned and intensity quantified using ImageJ software.

Enzyme Kinetics and Respiration Measurements

For citrate synthase activity, the conversion of 5,5’-dithio-bis-[2-nitrobenzoic acid] (DTNB) to thionitrobenzoic acid (TNB) in clarified cell lysates was monitored at 412 nm. Seahorse Technology was used to measure respiration in HeLa cells ±8 hr prior to CCCP or valinomycin treatment. For aconitase activity, $NADP^+$ reduction during the conversion of citrate to α-ketoglutarate was monitored at 340 nm. Respiratory complexes I, II–III, and IV activities were determined by measuring rotenone-sensitive NADH:Q_1 oxidoreduction (340 nm), antimycin A-sensitive succinate:cytochrome *c* oxidoreduction (550 nm), and reduced cytochrome *c* oxidation (550 nm), respectively.

Real-Time qPCR of mRNA

RNA was extracted from antimycin A-treated SH-SY5Y cells using the RNeasy kit (QIAGEN) and converted to cDNA by the QuantiTect reverse transcription kit (QIAGEN). The QuantiTect SYBR Green kit (QIAGEN) was used with QuantiTect Primer Assays (QIAGEN) to determine mRNA levels by real-time qPCR, and measurements were normalized using the transcript levels for β-actin. Experiments were carried out in triplicate, and each measurement also was performed in triplicate. Relative expression was calculated using the ΔC_T method.

Immunofluorescence

HeLa cells were seeded at 3×10^5 on 24-mm glass coverslips in six-well plates and cultured overnight. Following requisite CCCP treatment, cells were fixed in 4% paraformaldehyde, PBS washed, and solubilized in PBS-0.25% Triton X-100 (20 min, room temperature [RT]). Blocking was performed overnight in PBS-5% BSA (4°C). Anti-HSP60 and anti-NDUFV2 (both 500× in blocking buffer) were co-incubated for 90 min (RT), followed by anti-rabbit Alexa Fluor 594- and anti-mouse Alexa Fluor 488-conjugated secondary antibodies (500× in PBS) for 60 min (RT).

Figure 3. Activating Mitophagy Induces Mitochondrial Oxidative Stress and Triggers Peripheral Arm Degradation

(A and B) Aconitase activity in HeLa cells after antimycin A (Anti.A) ± oligomycin (Oligo), rotenone (Rot) ± Oligo. Red zone indicates activity attributable to the cytosolic isoform. (n = 4–5).

(C) HeLa cells ± 8-hr CCCP ± NAC (n = 3).

(D and E) Anti.A-treated SH-SY5Y cells ± cycloheximide (CHX) (n = 3).

(F) mRNA levels in SH-SY5Y cells ± Anti.A treatment (n = 3).

(G and H) NAD(P)H autofluorescence (a.u.) in mitochondrial-GFP (mt-GFP)-expressing SH-SY5Y cells during Anti.A treatment (n = 3).

(I and J) Rates (a.u.) of ROS in mt-GFP SH-SY5Y cells incubated with mitoSOX and treated with Anti.A (n = 3).

(K) NADH-induced H_2O_2 production in mitochondria isolated from HeLa cells ± 8-hr CCCP (n = 3). All data in the figure are presented as mean + SDM.

See also Figure S2.

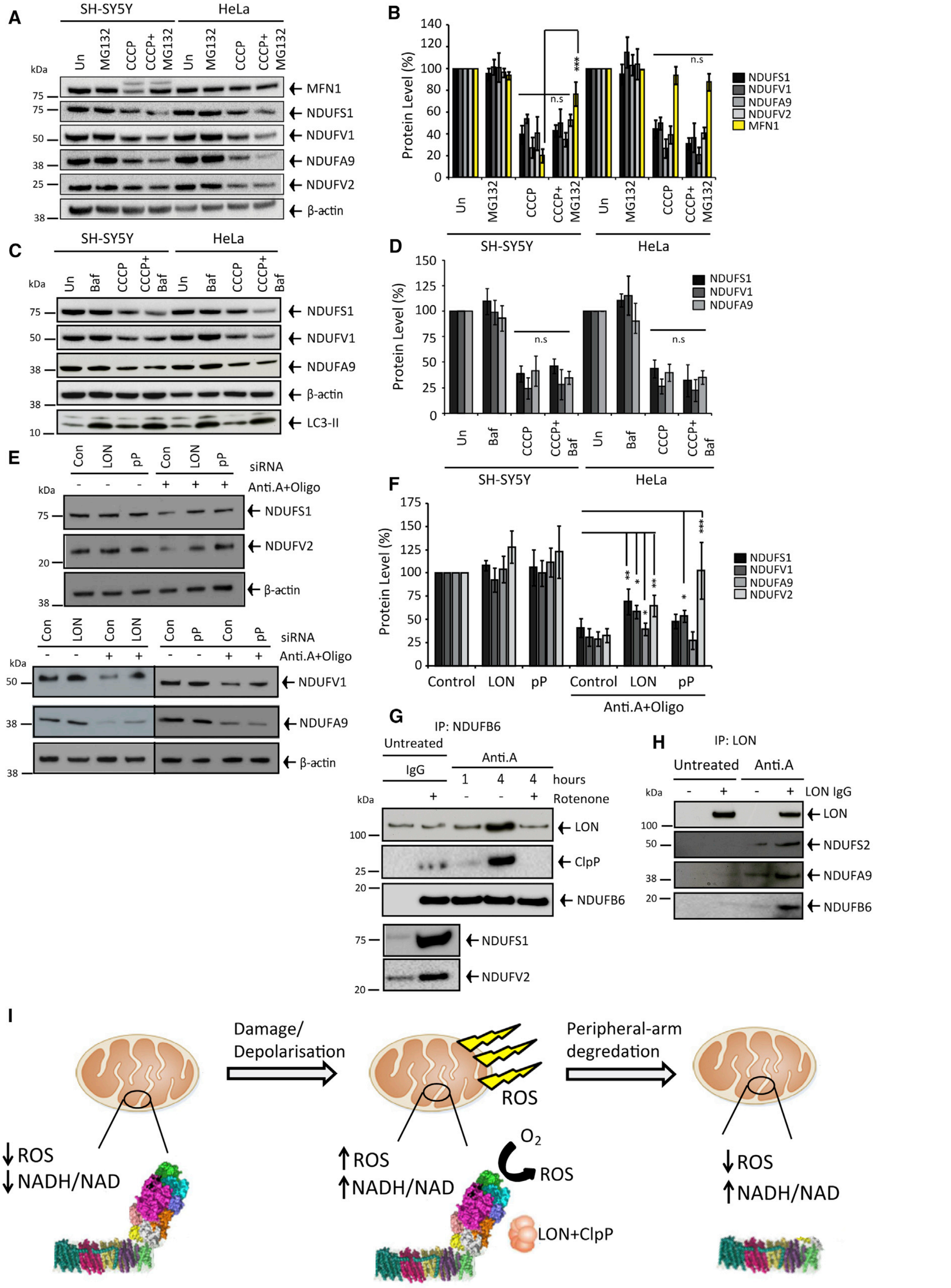

(legend on next page)

Live-Cell Imaging

SH-SY5Y cells expressing mt-GFP or HeLa cells were seeded onto 22-mm glass coverslips in six-well plates for 48 hr (1 × 10^5/well), and then they were loaded with 25 nM or 10 nM $TMRM^+$ (Thermo Fisher Scientific) (30 min in Hank's balanced salt solution (HBSS), RT). For ROS measurements, 20 μM MitoSOX (HBSS) was added for 10 min prior to rate measurements. NAD(P)H autofluorescence was excited at 351 nm and measured between 375 and 470 nm.

LON and ClpP Silencing

RNAi Max (Thermo Fisher Scientific) and Opti-MEM (Thermo Fisher Scientific) were used to silence 2 × 10^5 HeLa cells (according to the manufacturer's instructions) with 5 nM small interfering RNA (siRNA) LON (Thermo Fisher Scientific stealth siRNAs; HSS113887 and HSS113888), 5 nM siRNA ClpP (Thermo Fisher Scientific stealth siRNAs; HSS112033 and HSS112034), or 5 nM control siRNA (Thermo Fisher Scientific stealth siRNAs negative control) in six-well plates for 48 hr prior to treatment.

Complex I-LON and ClpP Co-immunoprecipitation

100 μL (~800–1,000 μg) of clarified PBS-1.5% n-Dodecyl β-D-Maltopyranoside lysates were incubated with recombinant protein G agarose (rPG agarose) (Life Technologies) for 45 min, subsequently sedimented, and supernatants were added to 30 μL sedimented rPG prior coated with anti-NDUFB6 (200×) or anti-LON (200×) for 2 hr (4°C). Beads were washed three times (PBS, 4°C), and antibody-antigen conjugates were eluted in 0.1 M glycine (pH 2.5, Life Technologies) and neutralized by 1M Tris. DTT-reduced, 70°C-heated samples were resolved by SDS-PAGE and antigens were detected by immunoblot.

Statistical Analysis

All data are expressed as the mean + SD of the mean (SDM). Statistical significance was calculated by using Student's t test, the nonparametric Kruskal-Wallis test (comparing more than two datasets), or the nonparametric Mann-Whitney test (comparing two datasets) on at least triplicate experiments (see figure legends). Considered significant, $p < 0.05$ is denoted with a single asterisk whereas $p < 0.01$ and $p < 0.001$ are denoted with two and three asterisks, respectively.

SUPPLEMENTAL INFORMATION

Supplemental Information includes Supplemental Experimental Procedures and three figures and can be found with this article online at http://dx.doi.org/10.1016/j.celrep.2016.11.027.

AUTHOR CONTRIBUTIONS

K.R.P. wrote the manuscript and performed, analyzed, and designed the experiments. J.W.T. supervised, performed, and designed experiments and wrote the manuscript. A.H.S. supervised, designed the experiments, and wrote the manuscript.

ACKNOWLEDGMENTS

We thank Professor John Hardy for PD-PINK1 fibroblasts. This study was supported by the Wellcome Trust/Medical Research Council (MRC) Joint Call in Neurodegeneration award (WT089698) and Centre of Excellence in Neurodegeneration award (MR/J009660/1). A.H.S. is supported by the UCLH NIHR BRC and is an NIHR Senior Investigator.

Received: March 7, 2016
Revised: September 28, 2016
Accepted: November 3, 2016
Published: December 6, 2016

REFERENCES

Almeida, A., Almeida, J., Bolaños, J.P., and Moncada, S. (2001). Different responses of astrocytes and neurons to nitric oxide: the role of glycolytically generated ATP in astrocyte protection. Proc. Natl. Acad. Sci. USA *98*, 15294–15299.

Babot, M., Labarbuta, P., Birch, A., Kee, S., Fuszard, M., Botting, C.H., Wittig, I., Heide, H., and Galkin, A. (2014). ND3, ND1 and 39kDa subunits are more exposed in the de-active form of bovine mitochondrial complex I. Biochim. Biophys. Acta *1837*, 929–939.

Bender, T., Lewrenz, I., Franken, S., Baitzel, C., and Voos, W. (2011). Mitochondrial enzymes are protected from stress-induced aggregation by mitochondrial chaperones and the Pim1/LON protease. Mol. Biol. Cell *22*, 541–554.

Bota, D.A., and Davies, K.J. (2002). Lon protease preferentially degrades oxidized mitochondrial aconitase by an ATP-stimulated mechanism. Nat. Cell Biol. *4*, 674–680.

Chinta, S.J., Kumar, M.J., Hsu, M., Rajagopalan, S., Kaur, D., Rane, A., Nicholls, D.G., Choi, J., and Andersen, J.K. (2007). Inducible alterations of glutathione levels in adult dopaminergic midbrain neurons result in nigrostriatal degeneration. J. Neurosci. *27*, 13997–14006.

Chouchani, E.T., Methner, C., Nadtochiy, S.M., Logan, A., Pell, V.R., Ding, S., James, A.M., Cochemé, H.M., Reinhold, J., Lilley, K.S., et al. (2013). Cardioprotection by S-nitrosation of a cysteine switch on mitochondrial complex I. Nat. Med. *19*, 753–759.

Chung, K.K., Zhang, Y., Lim, K.L., Tanaka, Y., Huang, H., Gao, J., Ross, C.A., Dawson, V.L., and Dawson, T.M. (2001). Parkin ubiquitinates the alpha-synuclein-interacting protein, synphilin-1: implications for Lewy-body formation in Parkinson disease. Nat. Med. *7*, 1144–1150.

Danielson, S.R., Held, J.M., Oo, M., Riley, R., Gibson, B.W., and Andersen, J.K. (2011). Quantitative mapping of reversible mitochondrial Complex I cysteine oxidation in a Parkinson disease mouse model. J. Biol. Chem. *286*, 7601–7608.

Galkin, A., Meyer, B., Wittig, I., Karas, M., Schägger, H., Vinogradov, A., and Brandt, U. (2008). Identification of the mitochondrial ND3 subunit as a structural component involved in the active/deactive enzyme transition of respiratory complex I. J. Biol. Chem. *283*, 20907–20913.

Gardner, P.R., Raineri, I., Epstein, L.B., and White, C.W. (1995). Superoxide radical and iron modulate aconitase activity in mammalian cells. J. Biol. Chem. *270*, 13399–13405.

Gorenkova, N., Robinson, E., Grieve, D.J., and Galkin, A. (2013). Conformational change of mitochondrial complex I increases ROS sensitivity during ischemia. Antioxid. Redox Signal. *19*, 1459–1468.

Grivennikova, V.G., Maklashina, E.O., Gavrikova, E.V., and Vinogradov, A.D. (1997). Interaction of the mitochondrial NADH-ubiquinone reductase with rotenone as related to the enzyme active/inactive transition. Biochim. Biophys. Acta *1319*, 223–232.

Kane, L.A., Lazarou, M., Fogel, A.I., Li, Y., Yamano, K., Sarraf, S.A., Banerjee, S., and Youle, R.J. (2014). PINK1 phosphorylates ubiquitin to activate Parkin E3 ubiquitin ligase activity. J. Cell Biol. *205*, 143–153.

Figure 4. LON and ClpP Bind Intact Complex I and Mediate Peripheral Arm Degradation

(A–D) SH-SY5Y and HeLa cells ± 8-hr MG132 (n = 3) or bafilomycin (Baf) (n = 3) ± CCCP.

(E and F) Control (n = 4), LON (n = 3), or ClpP (n = 3) silenced HeLa cells ± 8-hr antimycin A (Anti.A) + oligomycin (Oligo).

(G and H) NDUFB6 precipitation with ClpP (n = 3) and LON (n = 3) and LON precipitation with complex I (n = 3) ± Anti.A ± rotenone.

(I) Cartoon model of proteolytic quality control in depolarized mitochondria to extinguish ROS production. All data in the figure are presented as mean + SDM. See also Figure S3.

Keeney, P.M., Xie, J., Capaldi, R.A., and Bennett, J.P., Jr. (2006). Parkinson's disease brain mitochondrial complex I has oxidatively damaged subunits and is functionally impaired and misassembled. J. Neurosci. *26*, 5256–5264.

Kim, H., Lee, H., and Shin, D. (2015). Lon-mediated proteolysis of the FeoC protein prevents *Salmonella enterica* from accumulating the Fe(II) transporter FeoB under high-oxygen conditions. J. Bacteriol. *197*, 92–98.

Kitada, T., Asakawa, S., Hattori, N., Matsumine, H., Yamamura, Y., Minoshima, S., Yokochi, M., Mizuno, Y., and Shimizu, N. (1998). Mutations in the parkin gene cause autosomal recessive juvenile parkinsonism. Nature *392*, 605–608.

Koopman, W.J., Nijtmans, L.G., Dieteren, C.E., Roestenberg, P., Valsecchi, F., Smeitink, J.A., and Willems, P.H. (2010). Mammalian mitochondrial complex I: biogenesis, regulation, and reactive oxygen species generation. Antioxid. Redox Signal. *12*, 1431–1470.

Kotlyar, A.B., and Vinogradov, A.D. (1990). Slow active/inactive transition of the mitochondrial NADH-ubiquinone reductase. Biochim. Biophys. Acta *1019*, 151–158.

Kraytsberg, Y., Kudryavtseva, E., McKee, A.C., Geula, C., Kowall, N.W., and Khrapko, K. (2006). Mitochondrial DNA deletions are abundant and cause functional impairment in aged human substantia nigra neurons. Nat. Genet. *38*, 518–520.

Kushnareva, Y., Murphy, A.N., and Andreyev, A. (2002). Complex I-mediated reactive oxygen species generation: modulation by cytochrome c and NAD(P)+ oxidation-reduction state. Biochem. J. *368*, 545–553.

Lambert, A.J., Buckingham, J.A., Boysen, H.M., and Brand, M.D. (2010). Low complex I content explains the low hydrogen peroxide production rate of heart mitochondria from the long-lived pigeon, Columba livia. Aging Cell *9*, 78–91.

Malhotra, K., Sathappa, M., Landin, J.S., Johnson, A.E., and Alder, N.N. (2013). Structural changes in the mitochondrial Tim23 channel are coupled to the proton-motive force. Nat. Struct. Mol. Biol. *20*, 965–972.

Maklashina, E., Sher, Y., Zhou, H.Z., Gray, M.O., Karliner, J.S., and Cecchini, G. (2002). Effect of anoxia/reperfusion on the reversible active/de-active transition of NADH0ubiquinone oxidoreductase (complex I) in rat heart. Biochim Biophys Acta *1556*, 6–12.

Miwa, S., Jow, H., Baty, K., Johnson, A., Czapiewski, R., Saretzki, G., Treumann, A., and von Zglinicki, T. (2014). Low abundance of the matrix arm of complex I in mitochondria predicts longevity in mice. Nat. Commun. *5*, 3837.

Morais, V.A., Haddad, D., Craessaerts, K., De Bock, P.J., Swerts, J., Vilain, S., Aerts, L., Overbergh, L., Grünewald, A., Seibler, P., et al. (2014). PINK1 loss-of-function mutations affect mitochondrial complex I activity via NdufA10 ubiquinone uncoupling. Science *344*, 203–207.

Münch, C., and Harper, J.W. (2016). Mitochondrial unfolded protein response controls matrix pre-RNA processing and translation. Nature *534*, 710–713.

Narendra, D.P., Jin, S.M., Tanaka, A., Suen, D.F., Gautier, C.A., Shen, J., Cookson, M.R., and Youle, R.J. (2010). PINK1 is selectively stabilized on impaired mitochondria to activate Parkin. PLoS Biol. *8*, e1000298.

Ngo, J.K., and Davies, K.J. (2009). Mitochondrial Lon protease is a human stress protein. Free Radic. Biol. Med. *46*, 1042–1048.

Nishii, W., Suzuki, T., Nakada, M., Kim, Y.T., Muramatsu, T., and Takahashi, K. (2005). Cleavage mechanism of ATP-dependent Lon protease toward ribosomal S2 protein. FEBS Lett. *579*, 6846–6850.

Ondrovicová, G., Liu, T., Singh, K., Tian, B., Li, H., Gakh, O., Perecko, D., Janata, J., Granot, Z., Orly, J., et al. (2005). Cleavage site selection within a folded substrate by the ATP-dependent lon protease. J. Biol. Chem. *280*, 25103–25110.

Parker, W.D., Jr., Parks, J.K., and Swerdlow, R.H. (2008). Complex I deficiency in Parkinson's disease frontal cortex. Brain Res. *1189*, 215–218.

Pryde, K.R., and Hirst, J. (2011). Superoxide is produced by the reduced flavin in mitochondrial complex I: a single, unified mechanism that applies during both forward and reverse electron transfer. J. Biol. Chem. *286*, 18056–18065.

Quirós, P.M., Langer, T., and López-Otín, C. (2015). New roles for mitochondrial proteases in health, ageing and disease. Nat. Rev. Mol. Cell Biol. *16*, 345–359.

Radke, S., Chander, H., Schäfer, P., Meiss, G., Krüger, R., Schulz, J.B., and Germain, D. (2008). Mitochondrial protein quality control by the proteasome involves ubiquitination and the protease Omi. J. Biol. Chem. *283*, 12681–12685.

Schägger, H. (1995). Quantification of oxidative phosphorylation enzymes after blue native electrophoresis and two-dimensional resolution: normal complex I protein amounts in Parkinson's disease conflict with reduced catalytic activities. Electrophoresis *16*, 763–770.

Schapira, A.H. (2008). Mitochondria in the aetiology and pathogenesis of Parkinson's disease. Lancet Neurol. *7*, 97–109.

Schapira, A.H., Cooper, J.M., Dexter, D., Jenner, P., Clark, J.B., and Marsden, C.D. (1989). Mitochondrial complex I deficiency in Parkinson's disease. Lancet *1*, 1269.

Soubannier, V., McLelland, G.L., Zunino, R., Braschi, E., Rippstein, P., Fon, E.A., and McBride, H.M. (2012). A vesicular transport pathway shuttles cargo from mitochondria to lysosomes. Curr. Biol. *22*, 135–141.

Valente, E.M., Abou-Sleiman, P.M., Caputo, V., Muqit, M.M., Harvey, K., Gispert, S., Ali, Z., Del Turco, D., Bentivoglio, A.R., Healy, D.G., et al. (2004). Hereditary early-onset Parkinson's disease caused by mutations in PINK1. Science *304*, 1158–1160.

Varghese, F., Atcheson, E., Bridges, H.R., and Hirst, J. (2015). Characterization of clinically identified mutations in NDUFV1, the flavin-binding subunit of respiratory complex I, using a yeast model system. Hum. Mol. Genet. *24*, 6350–6360.

Zhu, J., Vinothkumar, K.R., and Hirst, J. (2016). Structure of mammalian respiratory complex I. Nature *536*, 354–358.

Cell Reports, Volume *17*

Supplemental Information

A LON-ClpP Proteolytic Axis Degrades Complex I to Extinguish ROS Production in Depolarized Mitochondria

Kenneth Robert Pryde, Jan Willem Taanman, and Anthony Henry Schapira

Supplementary information

Supplementary Figure 1

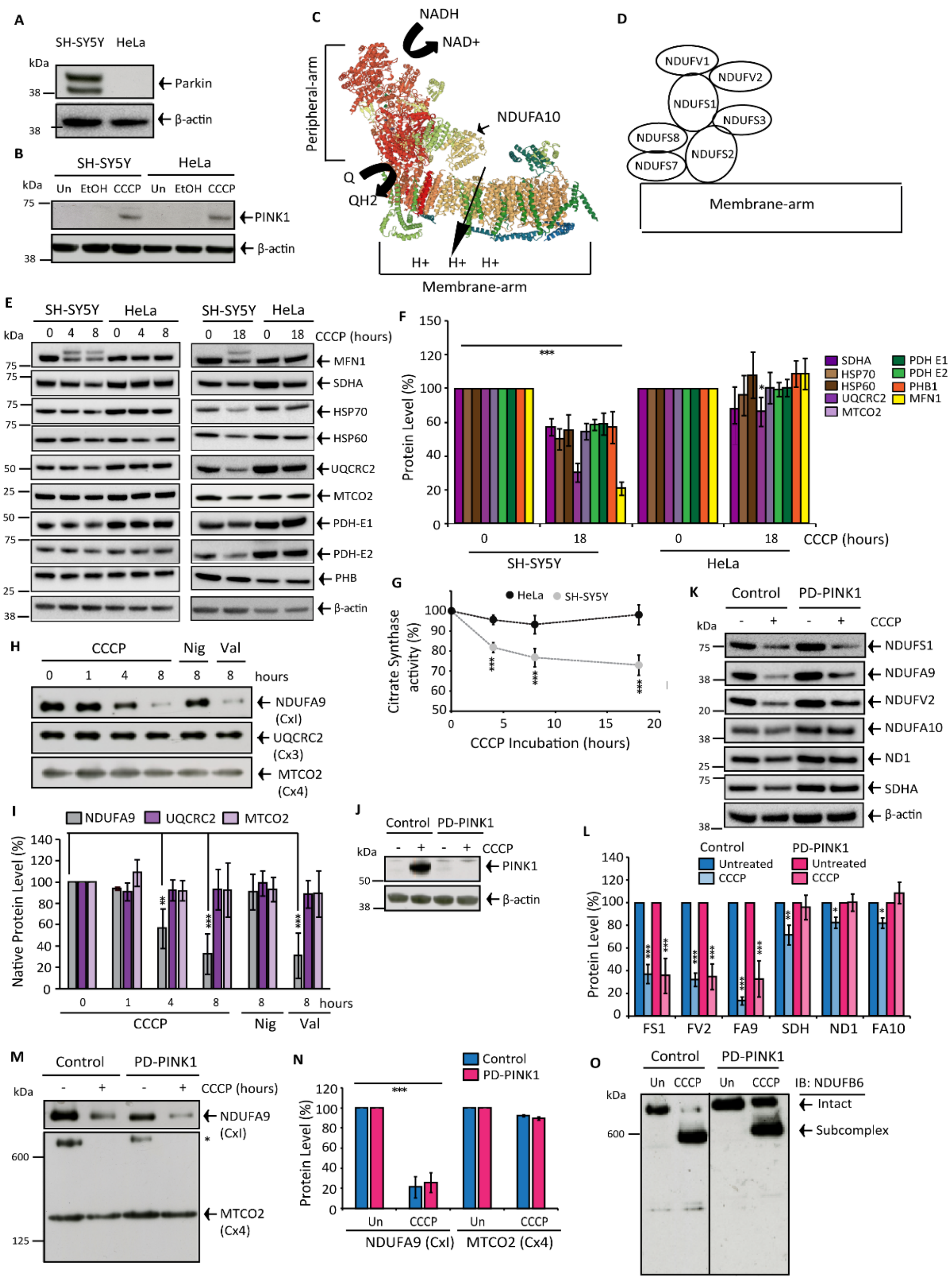

Supplementary Figure 1. Related to Figure 1: Complex I architecture, mitochondrial marker protein abundance during CCCP treatment and complex I degradation in control/PD-PINK1 fibroblasts. **(A-B)** SH-SY5Y and HeLa cells ± 18 hours CCCP or ethanol (EtOH). **(C)** High resolution structure of complex I from *Bos taurus* determined by single-particle cryo-microscopy. Protein Data Bank code 4UQ8.pdb. **(D)** Cartoon depiction of peripheral-arm subunit configuration. **(E-F)** SH-SY5Y and HeLa cells ± CCCP treatment (n=4-5). **(G)** Citrate synthase activity in SH-SY5Y and HeLa cells ± CCCP. **(H-I)** Native-PAGE. SH-SY5Y cells ± CCCP, nigericin (Nig) or valinomycin (Val) (n=3). **(J)** Control and PD-PINK1 fibroblasts ± 18 hours CCCP. **(K-L)** Control and PD-PINK1 fibroblasts ± CCCP (n=4). **(M-N)** Native-PAGE. Control and PD-PINK1 fibroblasts ± 18 hours CCCP. * indicates remnant complex I staining (n=3). **(O)** Native-PAGE. Control and PINK1-PD fibroblasts ± 18 hours CCCP. All data presented as mean + SDM.

Supplementary Figure 2

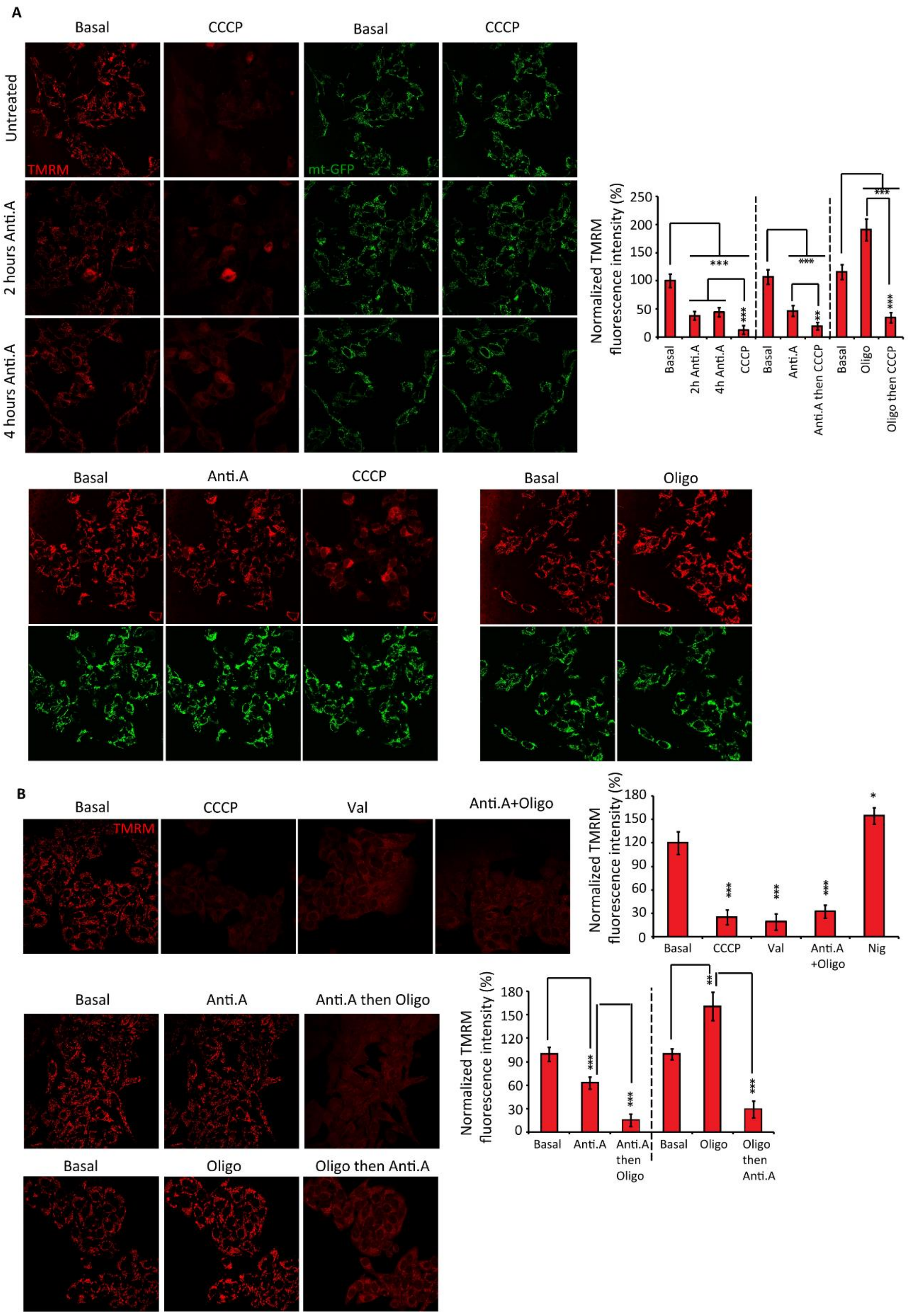

Supplementary Figure 2. Related to Figure 2: Effect of mitochondrial toxins on TMRM$^+$ fluorescence. **(A-B)** TMRM$^+$ fluorescence in SH-SY5Y cells expressing mitochondrial-targeted GFP **(A)** or HeLa cells **(B)**. CCCP, oligomycin (Oligo), valinomycin (Val), nigericin (Nig), antimycin A (Anti.A) and Anti.A+Oligo added as indicated. Representative images are taken from the steady-states for respective conditions (n=3). In **(B)** the upper panels/images reflect the average steady-state fluorescence after toxin addition in separate experiments (e.g. Val was not added after CCCP). All data presented in figure are mean values + SDM.

Supplementary Figure 3

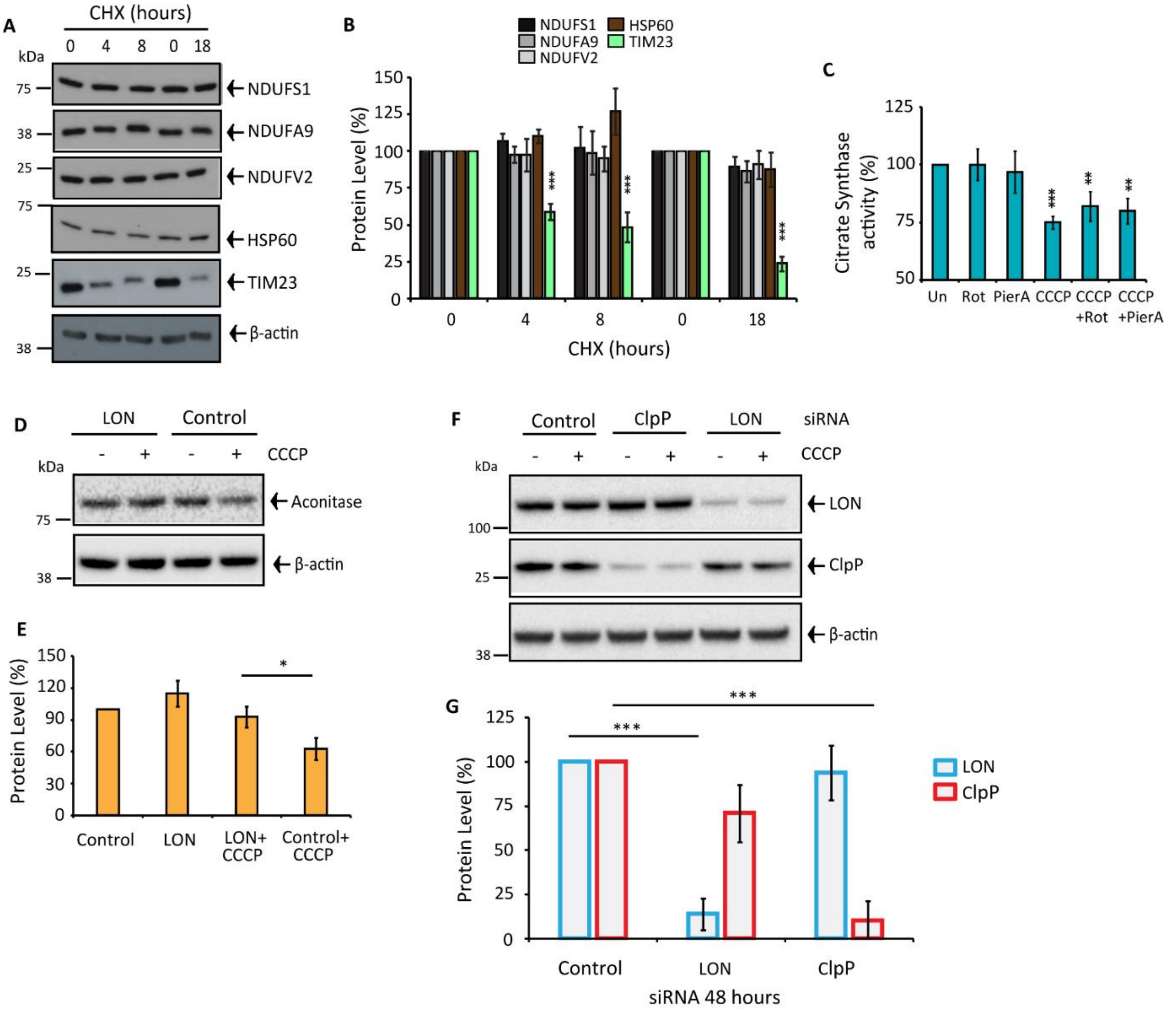

Supplementary Figure 3. Related to Figures 2, 3 and 4: Cycloheximide does not affect complex I level and the LON/ClpP silencing efficacy.
(A-B) HeLa cells ± cycloheximide (CHX) (n=3). **(C)** Citrate synthase activity. SH-SY5Y cells ±18 hours CCCP ± rotenone (Rot) or piericidin A (Pier.A) (n=4). **(D-E)** Aconitase immunoblots and quantification. HeLa cells ± 48 hours LON silencing followed by 8 hours CCCP (n=3). **(F-G)** ClpP (n=4) and LON (n=4) silencing in HeLa cells (48 hours) ± 8 hours CCCP. All data presented in figure are mean values + SDM.

Reagents and antibodies

All reagents/chemicals were purchased from Sigma-Aldrich unless otherwise stated. Antibodies were as follows: rabbit anti-NDUFS1 (Abcam - ab169540), rabbit anti-NDUFV1 (ProteinTech - 11238-1-AP), mouse anti-NDUFS2 (Abcam - ab192022), mouse anti-NDUFA9 (Abcam - ab14713), rabbit anti-NDUFV2 (ProteinTech - 15301-1-AP), mouse anti-NDUFB6 (Abcam - ab110244), mouse anti-NDUFB8 (Abcam - ab110242), rabbit anti-ND1 (ProteinTech - 19703-1-AP), rabbit anti-NDUFA10 (Abcam - ab174829), mouse anti-MFN1 (Abcam - ab57602), mouse anti-SDHA (Abcam - ab14715), mouse anti-UQCRC2 (Abcam - ab203832), mouse anti-PDH E1 (ProteinTech - 66119-1-Ig), rabbit anti-PDHA2 (Abcam - ab107015), mouse anti-β-actin (Abcam - ab8226), mouse anti-HSP60 (BioLegend – 681502), mouse anti-HSP70 (BioLegend - 648002), rabbit anti-PHB1 (Cell Signaling – 2426S), rabbit anti-LONP1 (ProteinTech - 15440-1-AP), rabbit anti-ClpP (Abcam - ab124822), rabbit anti-LC3B (D11) (Cell Signaling - 3868), rabbit anti-PINK1 (Novus Biologicals - BC100-494), mouse anti-Parkin (Santa Cruz Biotechnology - sc-32282), mouse anti-MTCO2 (Abcam - ab110258), mouse anti-TIM23 (BD Biosciences - 611222); rabbit anti-aconitase2 (ProteinTech 11134-1-AP).

Cell treatments

20 µM CCCP was added to SH-SY5Y or HeLa (S3) cells and 40 µM added to fibroblasts. When required, 1 µM valinomycin (Abcam), 0.5 µM nigericin (Abcam), 10 µM rotenone (Abcam), 10 µM piericidin A (Cayman Chemicals), 10 µM antimycin A (Abcam), 5 µM oligomycin (Abcam), 10 mM NAC (Sigma), 25 µM MG-132 (Abcam), 0.2 µM bafilomycin (Abcam) and 50 µg mL^{-1} cycloheximide (Sigma) was used.

Human primary fibroblasts

Control fibroblasts were from a 52 year old male and the PD-PINK1 deficient fibroblasts were cultured from a 48 year old male carrying a homozygous frameshift deletion in PINK1 (c.261_276del16;p.Try90Leufsx12).

Blue native-PAGE and 'in-gel' complex I activity measurements

Cell pellets were solubilised in 50 µL 1 M 6-aminocaproic acid, 50 mM bistris (pH 7 (HCl), 1.5 % n-Dodecyl β-D-Maltopyranoside (Anatrace) supplemented with 1 mM PMSF, 1 µg mL^{-1} of pepstatin and 1 µg mL^{-1} of leupeptin. Clarified lysates were added to Serva blue G loading buffer (5 % Serva blue G in 1 M 6-aminocaproic acid) with approximately 20-25 µg protein loaded onto non-commercial 3-12% acrylamide gels (acrylamide:bis-acrylamide, 0.5 mM 6-aminocaproic acid, 50 mM bistris (pH 7)) (Schägger, 1995). Complexes were resolved at 4 mA/gel for approximately 70 minutes. Cathode buffer comprised 50 mM tricine, 15 mM bistris, 0.02 % Serva blue G and the anode buffer 50 mM bistris. For immunoblot analysis, wet transfers were performed at 100 V for 1 hour and membranes subsequently processed as standard.

For 'in-gel' activity measurements the cathode buffer was replaced after 12 minutes with 50 mM tricine, 15 mM bistris without Serva blue. After ~ 65 minutes the gels were stained with a 2 mM Tris (pH 7.4, HCl), 150 µM NADH and 3 mM nitroblue tetrazolium for 1-2 hours at 37 ºC. The assay buffer was exchanged with 10 % acetic acid-40 % methanol followed by distilled water. Gels were scanned and intensity quantified using Image J software.

Mitochondrial isolation

HeLa cells were re-suspended in ice cold 250 mM sucrose, 1 mM EDTA, 10 mM Tris (pH 7.4 (HCl)) buffer supplemented with 1x EDTA-free protease-phosphatase inhibitors, ruptured via mechanical homogenisation (1500 RPM, 20 strokes) and then pelleted (1500 g, 10 mins, 4 ºC). Supernatants were collected and pellets re-homogenised (1500 RPM, 20 strokes), subsequently clarified (at 1500 g, 10 mins, 4 ºC) and supernatants pooled. Mitochondria were pelleted (11800 g, 12 minutes, 4 ºC), twice washed in isolation buffer, and pellets finally re-suspended in 50-125 µL isolation buffer.

Citrate synthase, aconitase, respiratory complexes I-IV and H_2O_2 activity measurements

Citrate synthase activity: 10-40 µg mL^{-1} of 1 % Triton X-100-PBS clarified cell lysate was added to 200 µM DTNB, oxaloacetate and acetyl-CoA (50 mM Tris pH 8.0). The steady-state formation of TNB was measured at 412 nm.

Aconitase activity: 1-2 mg mL^{-1} of cell lysate or 0.1-0.25 mg mL^{-1} mitochondrial lysate was incubated with 200 µM $NADP^+$, 1 mM $MgCl_2$, 500 µM sodium citrate and exogenous isocitrate dehydrogenase (50 mM Tris pH 8.0). Steady-state rates of NADPH formation were monitored at 340 nm.

Respiratory complexes I-IV: Isolated mitochondria from cells ± CCCP treated were re-suspended in 320 mM sucrose, 1 mM K^+-EDTA, 10 mM Hepes (pH 7.4, HCl) and snap frozen 3x. Complex IV activity (550 nm) = 10 mM KPi buffer (pH 7.0) and 50 µM reduced cytochrome *c*, 30°C. Complexes II-III (550 nm) = 0.1 M KPi buffer (pH 7.4), 0.3 mM EDTA, 1 mM KCN, 20 mM succinate, 2.5 mg mL^{-1} BSA, 100 µM oxidised cytochrome *c* ± antimycin A, at 30°C. Complex I (340 nm) = 20 mM KPi buffer (pH 7.2), 1 mM $MgCl_2$, 1 mM KCN, 2.5 mg ml^{-1} BSA, 150 µM NADH, 50 µM CoQ_1 ± rotenone, 30°C.

Mitochondrial H_2O_2 production: Isolated mitochondria were snap frozen 3x (each time clarified (11800 g, 12 minutes, 4 °C)) and added to 80 µM NADH, 10 µM amplex red and 4 mg mL^{-1} HRP (250 mM sucrose, 10 mM Tris pH 7.5). Catalase-dependent steady-state rates of resorufin formation were monitored at 557 nm.

Respiration and glycolysis measurements in cultured cells

HeLa cells were seeded into XFp miniplates (Seahorse Bioscience) at $2.5x10^4$ and cultured overnight. Subsequently, cells were washed and incubated with XF base medium supplemented with exogenous 10 mM glucose, 1 mM pyruvate and 2 mM glutamine (pH 7.4 (NaOH)) and placed in a CO_2-free incubator at 37°C for 60 minutes. Oxygen consumption rates and extracellular acidification rates were measured in a XFp extracellular flux analyser (Seahorse, Agilent Technologies). Rates were adjusted if necessary following protein determination (BCA) and the antimycin A+rotenone insensitive rates were subtracted.

Obtaining and quantifying immunofluorescence and live-cell imaging data

For fixed cells images were acquired using an inverted Zeiss LSM 510 confocal microscope and a 63x oil-immersion objective. Alexa 594 was excited using the 543 nm laser line (HeNe laser) and fluorescence detected with a 560 nm long-pass filter. Alexa 488 was excited using a 488 nm laser line (Argon laser) and the fluorescence measured between 510 nm to 550 nm. Laser power and other settings were conserved during replicate experiments. Image J was used to quantify maximal fluorescence intensity (following background subtraction) in more than 15-25 cells per repeat experiment (n=3) and in 3-5 different fields of view. Data is presented as the average maximal fluorescence and in arbitrary units.

For live cell imaging GFP was excited using the 488 nm laser line (Argon laser) and emission measured between 510-550 nm, and $TMRM^+$ excited using the 543 nm laser line (HeNe laser) with emission measured using a 560 nm long-pass filter. MitoSOX was exited at 380 nm and emission followed using a 560 nm long-pass filter. NAD(P)H autofluorescence was excited at 351 nm and measured between 375-470 nm. Fields of view comprising 20-40 cells were selected and Z-projected (n = 3). Imaging was performed using a Zeiss LSM 510 (NAD(P)H autofluorescence and mitoSOX) or Nikon confocal microscope (GFP and $TMRM^+$) and either a 63x oil-immersion objective (GFP, mitoSOX and $TMRM^+$) or a 60x water-immersion objective (NAD(P)H). Average intensities were determined across the Z-stack throughout the time course (ImageJ). Data is presented as the percentage change from the initial measurement or fluorescence intensity (arbitrary units). All toxins were added in a HBSS solution and when necessary comprised the equivalent concentration of fluorescent dye.
